# Supplementary material for: Stacking Model‐Based Classifiers for Dealing With Multiple Sets of Noisy Labels
Source: Biom J. 2025 Mar 12;67(2):e70042. doi: 10.1002/bimj.70042 (PMC11898607; doi:10.1002/bimj.70042)
Supplement: Supplementary file 1 — Supporting Information [file BIMJ-67-e70042-s001.zip › density-based-ensemble-model-main/competitors_real_data/netlab-master/netlab-master/help/demev1.htm]

Netlab Reference Manual demev1


# demev1

## Purpose

Demonstrate Bayesian regression for the MLP.

## Synopsis

```
demev1
```

## Description

The problem consists an input variable `x` which sampled from a
Gaussian distribution, and a target variable `t` generated by
computing `sin(2*pi*x)` and adding Gaussian noise. A 2-layer
network with linear outputs is trained by minimizing a sum-of-squares
error function with isotropic Gaussian regularizer, using the scaled
conjugate gradient optimizer. The hyperparameters `alpha` and
`beta` are re-estimated using the function `evidence`. A graph
is plotted of the original function, the training data, the trained
network function, and the error bars.

## See Also

`evidence`, `mlp`, `scg`, `demard`, `demmlp1`

---

**Pages:**
Index


---

Copyright (c) Ian T Nabney (1996-9)
